# Climate change effect on wheat phenology depends on cultivar change

**DOI:** 10.1038/s41598-018-23101-2

**Published:** 2018-03-20

**Authors:** Ehsan Eyshi Rezaei, Stefan Siebert, Hubert Hüging, Frank Ewert

**Affiliations:** 1 Institute of Crop Science and Resource Conservation, University of Bonn, Katzenburgweg 5, D-53115 Bonn, Germany; 2 Center for Development Research (ZEF), Walter-Flex-Straße 3, 53113 Bonn, Germany; 3 Leibniz Centre for Agricultural Landscape Research, Institute of Landscape Systems Analysis,, D-15374 Müncheberg, Germany; 4 Department of Crop Sciences, University of Göttingen, Von-Siebold-Strasse 8, 37075 Göttingen, Germany

## Abstract

Changing crop phenology is considered an important bio-indicator of climate change, with the recent warming trend causing an advancement in crop phenology. Little is known about the contributions of changes in sowing dates and cultivars to long-term trends in crop phenology, particularly for winter crops such as winter wheat. Here, we analyze a long-term (1952–2013) dataset of phenological observations across western Germany and observations from a two-year field experiment to directly compare the phenologies of winter wheat cultivars released between 1950 and 2006. We found a 14–18% decline in the temperature sum required from emergence to flowering for the modern cultivars of winter wheat compared with the cultivars grown in the 1950s and 1960s. The trends in the flowering day obtained from a phenology model parameterized with the field observations showed that changes in the mean temperature and cultivar properties contributed similarly to the trends in the flowering day, whereas the effects of changes in the sowing day were negligible. We conclude that the single-cultivar concept commonly used in climate change impact assessments results in an overestimation of winter wheat sensitivity to increasing temperature, which suggests that studies on climate change effects should consider changes in cultivars.

## Introduction

The warming trend observed over the past few decades in many regions^1^ has caused considerable changes in plant phenology^2^. For example, 78% of all plant phenological records analyzed for 21 European countries showed a significant advancement in the occurrence of phenological events for the period from 1971–2000^3^. Although the changes in the phenology of natural vegetation and forests correspond well with the warming pattern, the changes in the phenology observed for annual crops have been more diverse because the effects of climate change on crop phenology have interacted with the effects of changes in crop management, such as modified sowing dates and changing cultivars^4^.

Adjusting sowing dates and changing cultivars could also be a useful intended or unintended adaptation to climate change, particularly for summer crops^5^. For example, earlier sowing dates prolonged the growing period of maize in northeastern China by 4 to 21 days^6^. New cultivars of rice and wheat with higher thermal requirements and later maturity dates that were introduced in China during 1981–2009 expanded their growth season lengths and compensated for the advancement in crop phenology caused by increased temperatures^7,8^. The length of the growing season for cereals such as wheat increased by approximately 10 days in Finland due to a cultivar change^9^. Prolonging the reproductive phase of maize by approximately 4 days per decade is considered an effective adaptation strategy to climate change in northern China^10^, whereas a third of the observed impact of climate change on wheat was compensated by new cultivars grown on the Loess Plateau of China^11^. The results of a series of experiments showed that the vernalization requirement and photoperiod sensitivity of new cultivars of winter wheat were significantly smaller than those of old cultivars in the U.S. Great Plains region^12,13^.

Disentangling the effects of climate change, sowing dates and cultivars on crop phenology is difficult because information on the changes of cultivar properties related to their phenological development is very limited. Variety trials compare different cultivars, but only recent cultivars are usually tested. Changes in the development rates of cultivars cannot be detected in these trials. Therefore, phenology models that were applied in previous studies assumed no change in cultivar properties but used climate change and sowing date to simulate changes in crop phenology caused by a changing climate. Although rarely considered, the effects of cultivar changes can be derived indirectly as the difference between the observed changes in crop phenology and the trends simulated with the phenology model^14^. The accuracy of the results obtained by using such an approach is determined primarily by the parameterization of the phenology model used in the study. For crops such as winter wheat, the vernalization requirement and the sensitivity to photoperiod and temperature must also be considered but are often not known for specific cultivars, which introduces considerable uncertainty in the study results.

**Figure 1:**
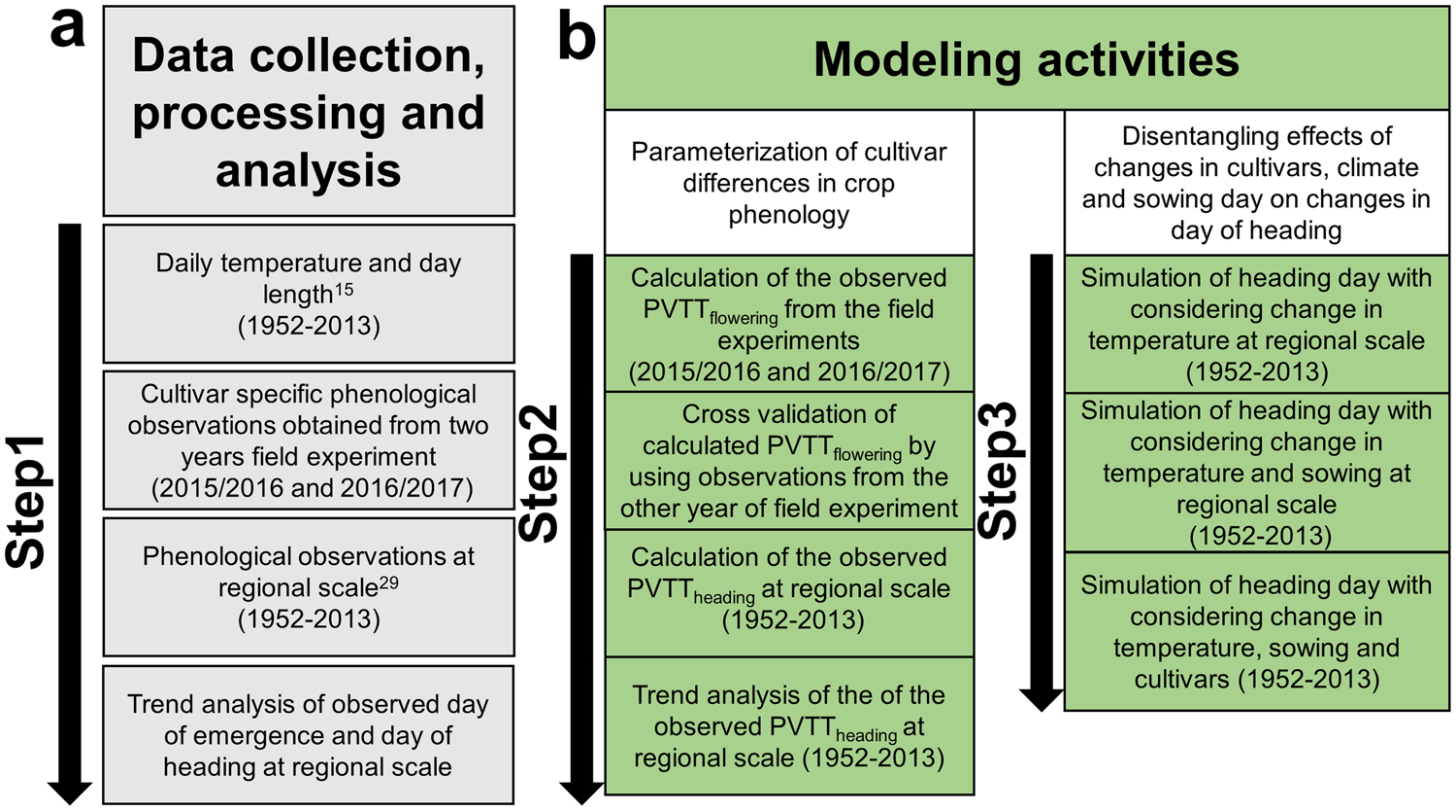
Overview of the workflow to prepare the climate and phenology data (**a**) and to develop the phenology model and to perform the analysis of the effect of changes in cultivars, climate and sowing dates on changes in the heading day (**b**).

The objective of the present study was to quantify the contributions of changes in cultivars, sowing dates and temperature to the changes in the phenology of winter wheat. In addition to long-term observations of crop phenology in western Germany, we parameterized a phenology model^15^ with cultivar-specific information obtained in a two-year variety trial by directly comparing historic and modern cultivars. We detected cultivar differences in the length of the period between emergence and flowering by growing 12 winter wheat varieties released over the past 60 years in Germany (Supplementary Table 1) in parallel at the Long-term Field Experiment Dikopshof near Cologne in western Germany. The selected cultivars represented popular varieties commonly grown in western Germany, typically for a period lasting approximately 10–15 years after their release. We applied the crop phenology model using the data collected in the two-year field experiment to calculate the cultivar-specific photo-vernal-thermal time (PVTT) required between emergence and flowering (PVTT_flowering_), which is the temperature sum corrected for the effects of photoperiod and vernalization. A piecewise linear regression was used to determine trends in the PVTT caused by the historical change in the cultivars. To test whether trends in the PVTT obtained from the cultivar experiment were representative for all of western Germany, we analyzed the long-term trends in the emergence dates and heading dates observed across the region for the period from 1952–2013 that were obtained from the German Meteorological Service and the derived trends in the PVTT from emergence to heading, and we compared the trends in the PVTTs obtained for the region with those caused by cultivar changes (Fig. 1). Subsequently, we used the crop phenology model, high-resolution climate data^15^ and sowing day observations^16^ to quantify the effect of changes in cultivars, sowing dates and temperature on the heading day of winter wheat. We focused on the effects of these factors on heading day because many crops are particularly sensitive to abiotic stresses such as drought, heat or frost in the period around heading or flowering.

## Results

### Long-term cultivar change (cultivar experiment)

**Figure 2:**
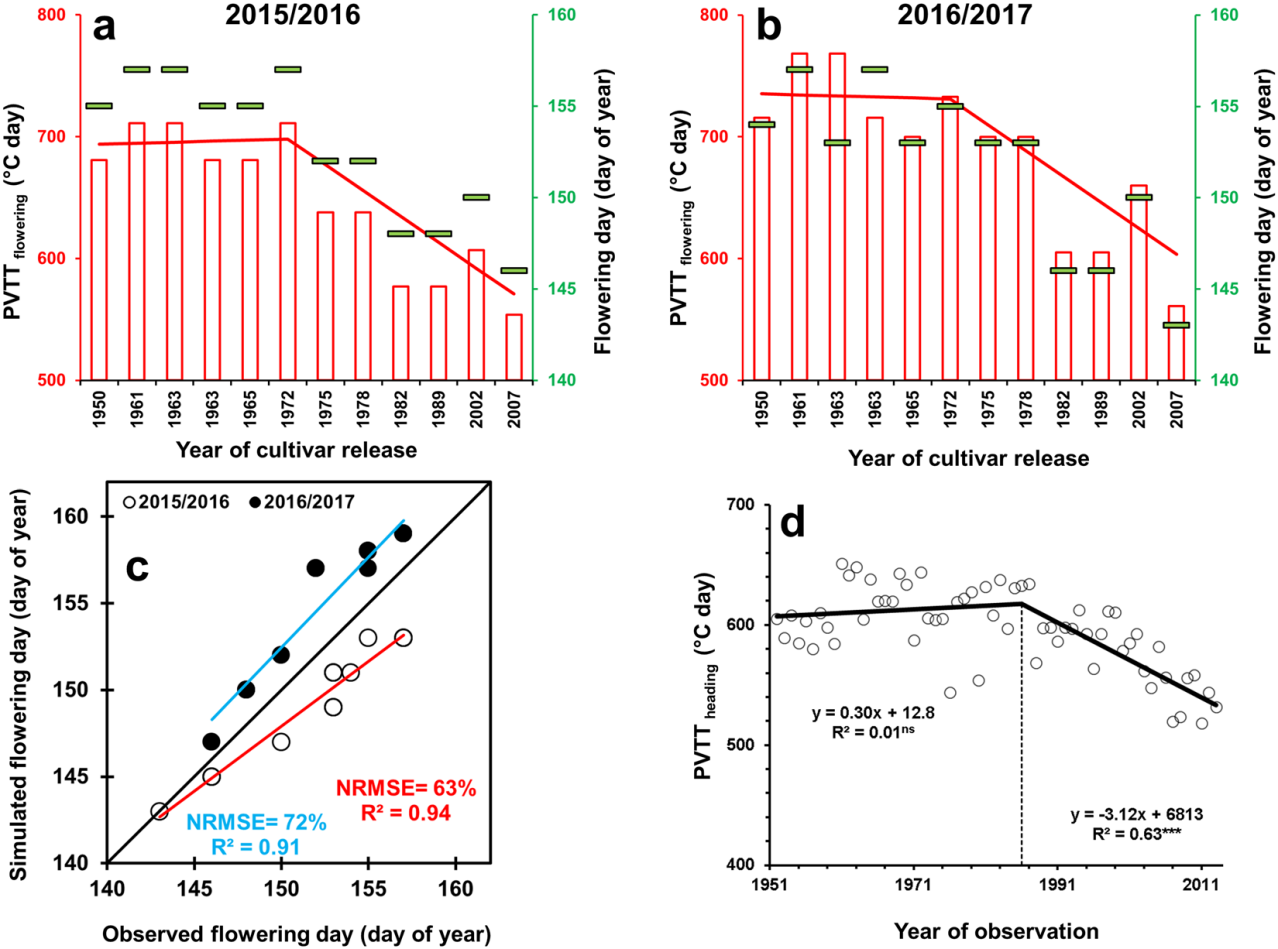
Cultivar-specific flowering day observed in the field experiment in 2015/2016 (**a**) and 2016/2017 (**b**); photo-vernal-thermal time (PVTT, °C day) from emergence to flowering calculated for 2015/2016 (**a**) and 2016/2017 (**b**); the 1:1 plot showing a cross validation of the phenology model by simulating the flowering day in 2016 by using the PVTT from 2016/2017 and simulating the flowering day in 2017 by using the PVTT from 2015/2016 (**c**); and the PVTT from emergence to heading (**d**) obtained from the long-term (1952–2013) phenology observations across western Germany.

The flowering time of the newest cultivar was 11 and 14 days earlier than the flowering time of older cultivars in first and second years of the field experiment, respectively (Fig. 2a,b). We also found that the PVTTs until flowering (PVTT_flowering_) of the new cultivars were 18% and 14% lower than the PVTT_flowering_ of the old cultivars in the first and second years of the field experiment, respectively, whereas the differences in the PVTT_flowering_ of the cultivars released before 1972 were small and did not show a clear trend (Fig. 2a,b). The cultivar-specific PVTT_flowering_ ranged from 554 °C day to 711 °C day and 560 °C day to 768 °C day based on the phenology observations obtained from the first and second years of the field experiment, respectively (Fig. 2a,b). If the other 11 parameters of the phenology model were well selected, then the PVTT_flowering_ should be a cultivar-specific constant, but we found that the PVTT_flowering_ of the same cultivars differed between the two years by up to 62 °C day or 9% (Fig. 2a,b). To quantify the corresponding uncertainties, we performed a cross validation by simulating the cultivar-specific flowering day in the specific years by using a cultivar-specific PVTT_flowering_ calculated for the other year and comparing the simulated flowering day with the observed flowering day (Fig. 2c).

We found that the model reproduced the differences in the cultivars very well based on the high coefficients of determination (R^2^ = 0.91 and 0.94 between the observed and simulated flowering day, respectively, Fig. 2c). The accuracy of the phenology model in estimating the exact flowering day was lower according to the normalized root mean square (NRMSE) values of 63% and 72% for the two seasons, respectively (Fig. 2c). However, we wanted to highlight that successfully reproducing the trend in the required PVTT of the cultivars is more important for answering our specific research questions than precise simulation of specific flowering days.

### Long-term changes in crop phenology (observer network western Germany)

The PVTT from emergence to heading (PVTT_heading_) calculated from the observations for western Germany showed similar trends, with a 15% smaller PVTT_heading_ in the most recent years than before 1986, which did not show a significant trend (Fig. 2d). Therefore, both the cultivar experiments and the regional phenology observations provided evidence that the PVTT_heading_ required for crop development in the vegetative phase declined considerably over the past three to four decades.

**Figure 3:**
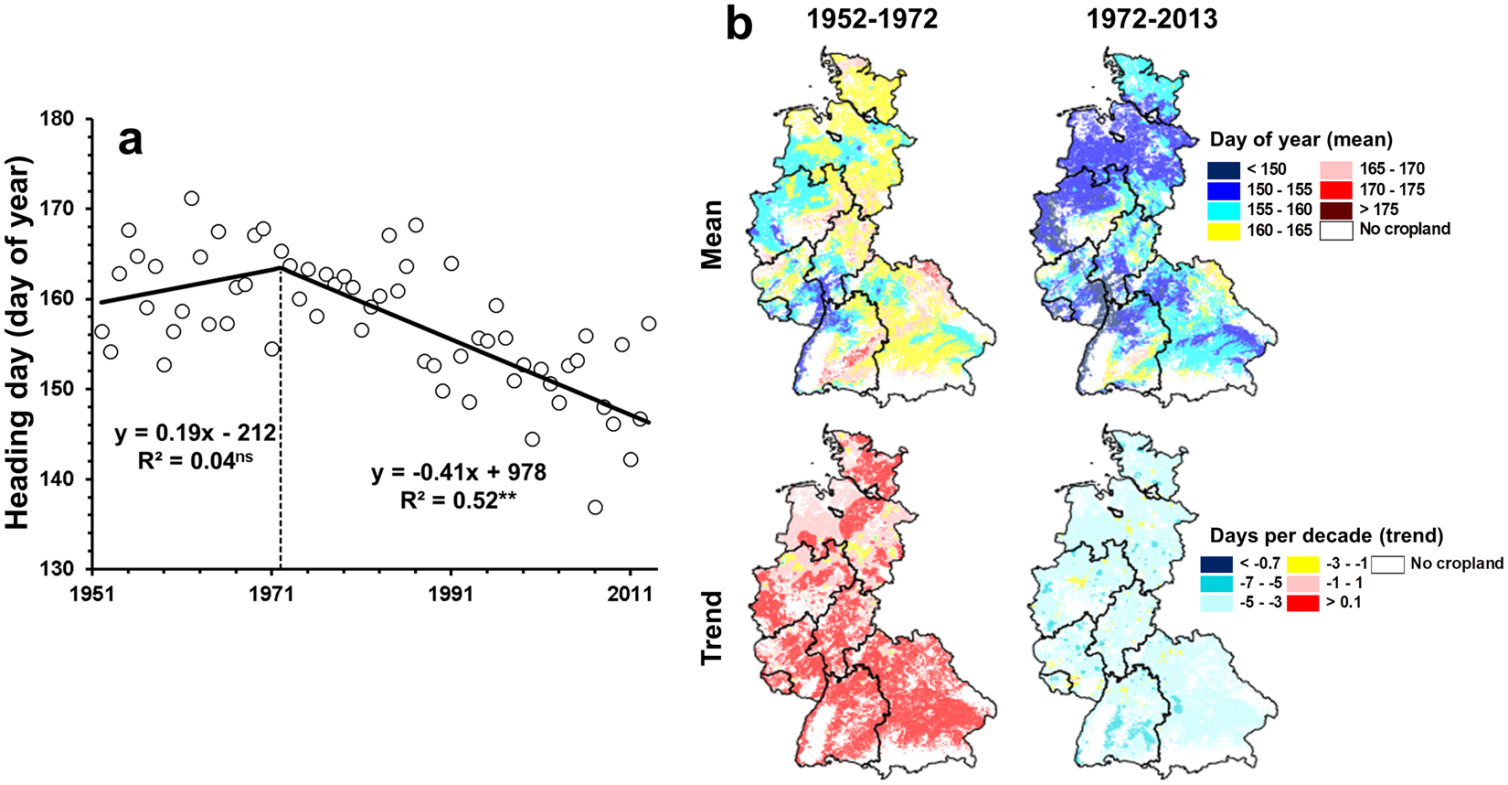
Trend (**a**) and spatial pattern (**b**) of the observed heading day in the period from 1952–2013 at a 1 km × 1 km resolution across western Germany. The spatial pattern indicated the mean and trend at the grid-cell level for the period before and after the break point in year 1972. The maps were generated with ArcGIS, version 10.3 (http://www.esri.com/arcgis) using shapefiles from the federal states obtained from the Federal Agency for Cartography and Geodesy of Germany (http://www.geodatenzentrum.de).

A significant negative trend (−4.1 days per decade) was detected for the heading day for the period 1972–2013 across western Germany, whereas the trend in heading day was not significant for the period before 1972 (Fig. 3). The negative trend in heading day showed a similar spatial pattern across the region for the period 1972–2013 (Fig. 3). Significant negative trends were also detected for the day of emergence and the length of the vegetative phase (emergence-heading) of winter wheat (Supplementary Figure 1). The trend of an earlier heading day of winter wheat in western Germany detected in the present study is consistent with the trends suggested in previous studies^16,17^.

### Contributions of the changes in cultivars, climate, and emergence date to changes in crop phenology (combined analysis)

**Figure 4:**
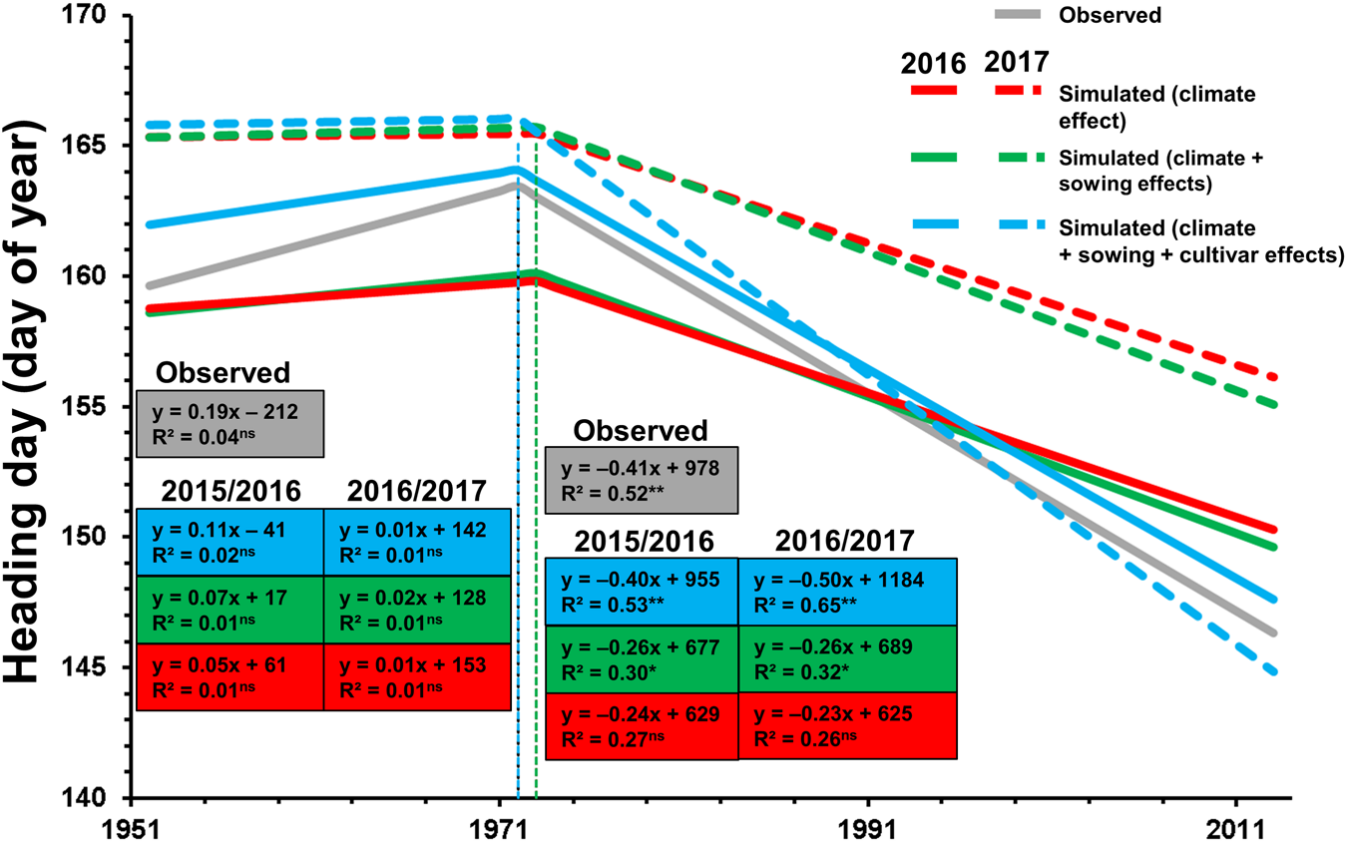
Mean heading day observed for western Germany and simulated with the phenology model (parametrized separately by field experiments conducted in 2015/2016 and 2016/2017) considering the climate effect (fixed sowing date and cultivars), combined climate and sowing effects (year- and grid-specific sowing date and fixed cultivar), and combined climate, sowing and cultivar effects (year and grid-specific date and changing cultivars) for the period from 1952 to 2013 for western Germany.

**Figure 5:**
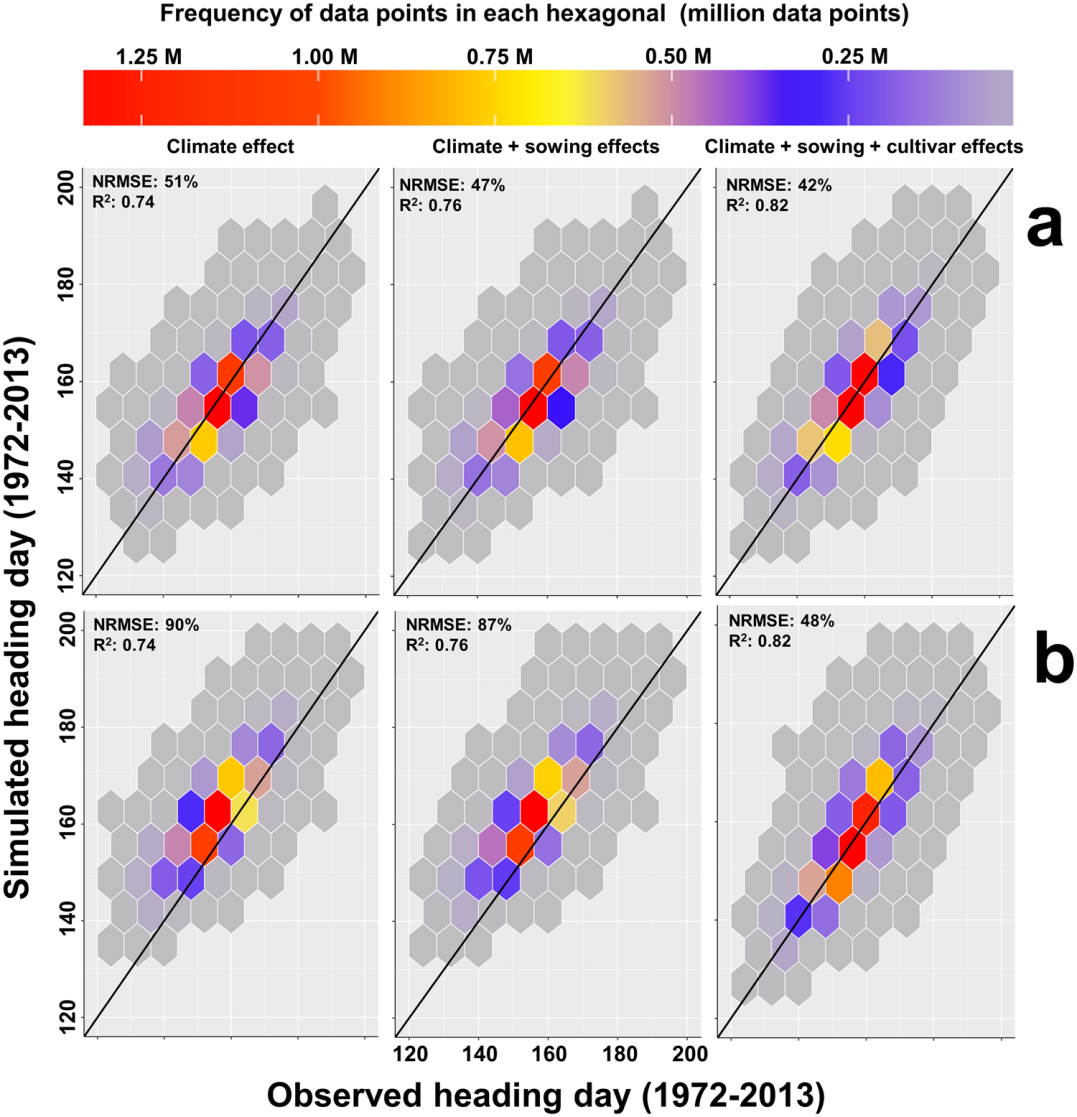
The 1:1 hexbin plot of the simulated (parameterized by the first (**a**) and second year (**b**) of the field experiment) and observed heading day based only on the climate effect, combined climate and sowing effects and combined climate, sowing and cultivar effects at a 1 km × 1 km resolution in the period 1972–2013 across western Germany. The color of each hexagonal cell shows the number of the simulated and observed points at each cell. Each panel of the figure included 12.5 million data points.

Temperature increases alone resulted in a negative but non-significant heading day trend of −2.3 to −2.4 days per decade (depending on the PVTT_heading_ used for the parameterization) in the heading day simulated for the period 1972–2013 for western Germany and therefore explained only half of the observed trend in heading day for that period, which was −4.1 days per decade (Fig. 4). Running the model with year and grid-specific sowing dates explained another (7%) of the trend in the simulated heading day (trend of −2.6 days per decade, Fig. 4). Running the model with a cultivar-dependent PVTT_heading_ for the specific cultivars derived from the field experiments resulted in a negative, significant heading day trend of −4.0 days per decade for the period 1972–2013, which reproduced the observed trend (−4.1 days per decade) almost exactly when using the cultivar-specific PVTT_heading_ from the 2015/2016 season (Fig. 4). Employing the cultivar-specific PVTT_heading_ using the data obtained in the cultivar experiment for the 2016/2017 season slightly overestimated the declining heading day trend (−5.0 days per decade simulated vs −4.1 days per decade observed, Fig. 4). The accuracy of the model simulations for the period from 1972–2013 where we found a negative trend in PVTT_heading_ also improved when we considered the combined effects of climate, sowing date and cultivar (NRMSE = 42% and 48%, R^2^ = 0.82) compared with simulations that only considered the effects of climate (NRMSE = 51% and 90%, R^2^ = 0.74) when using the PVTT_heading_ obtained from the 2015/2016 and 2016/2017 seasons, respectively (Fig. 5).

## Discussion

The advancement of the heading date observed for winter wheat in western Germany and the period from 1952–2013 was strongly associated with changes in the mean temperature in March-June (+0.5 °C per decade for the same period, Supplementary Figure 2). In previous studies, the negative trends in the occurrence of phenological stages of crops are exclusively explained by temperature increases without considering changes in cultivar properties^17–20^.

Our findings indicate that directly linking temperature increases to changes in phenology results in an overestimate of the sensitivity of crop phenology to changing temperatures for the region and crop studied here. Therefore, generally ignoring cultivar effects could introduce error in the climate impact assessment results. In most of the long-term impact assessment modeling studies performed at a large scale, crop phenology is parameterized based on field experiments that were conducted over the past 10 years, and a single parameter set is usually used to simulate crop phenology. Therefore, exploring changes in factors such as heat stress intensity or drought stress could be biased by the effects that cultivar changes have on crop phenology^21,22^.

We kept all the photoperiod and vernalization parameters in the phenology model constant and only changed the PVTT parameter to consider the effects of cultivar changes for long-term simulations. To test the suitability of this procedure, we performed a sensitivity analysis by adjusting the parameters related to the photoperiod and vernalization or the thermal requirements of the cultivars to reproduce days of flowering in the 2015/2016 season. Then, the parameter sets were applied to simulate flowering dates in the 2016/2017 season, and the simulated and observed days of flowering were compared. The results of the sensitivity analysis showed that phenology change is parameterized best by varying the thermal requirements of the cultivars, resulting in the lowest errors and a good reproduction of the trend in phenology (Supplementary Figure 3). A calibration based on the base photoperiod also reproduced the trend in phenology across the cultivars, but it was less precise in comparison to the calibration for the thermal requirements. In contrast, the calibration for vernalization days did not yield realistic parameters and was not able to reproduce the difference across the cultivars (Supplementary Figure 3). The phenology model was less precise when simulating the flowering dates of the old cultivars released before 1980 in the second year of the field experiment by using the PVTT obtained in the first year of the experiment, while the flowering days of the modern cultivars were well reproduced (Supplementary Figure 3a). This result indicates that the photoperiod and vernalization characteristics of the old cultivars differed from those of the modern cultivars. Therefore, the performance of the model can further be improved by adjusting the 2 photoperiod parameters in the model or the 6 parameters for vernalization. However, this calibration would require extended experimentation with different photoperiod and temperature combinations, which could be the objective of future research.

Separately utilizing the two datasets for the 2015/2016 and 2016/2017 seasons obtained from field experiments to parameterize the phenology model for the long-term simulations resulted in 5-day differences in the simulated heading day for both, simulations considering the climate effect and simulations considering climate and sowing day effects (Fig. 4). There was little difference in the trend for the simulated heading day parametrized by the two datasets over the last 62 years when we only considered climate and sowing effects after the breakpoint (Fig. 4). However, there was a difference of 1 day per decade between the trend of the heading day simulated by two sets of parameters when the combined climate, sowing and cultivar effects were considered in the period 1972–2013 (Fig. 4). The potential reason for this difference in the model results of the two parameter sets could be the limited number of cultivars used to establish the trend for the period 1972–2013 (seven cultivars). Specifically, the most recent cultivar Premio showed very early flowering in the 2016/2017 season (Fig. 2a,b), which influenced the trend for the whole period. Repeating the experiment with more cultivars is therefore suggested to obtain more robust trend estimates.

More research is needed to determine whether our findings can be generalized for other regions. Other research has shown that the required temperature sum for the flowering of modern durum wheat cultivars grown in Italy was significantly smaller than that for the old cultivars due to the reduction in photoperiod sensitivity and cold requirement^23^. The photoperiod sensitivity allele *Ppd-B1b* was found in 89% of the genotypes released before 1960 but declined to 52% in the genotypes released after 1999 in U.S. Great Plains region^12^. The faster development of new cultivars during the vegetative phase could also reduce heat and drought stress during the flowering period by shifting the flowering date to the cooler part of the growing season^16^. However, plant breeders are also facing other challenges such as avoiding crop damage due to frost events in the late spring when introducing early-flowering cultivars, specifically for western European conditions. Frost events that occur after the reproductive phase beings could result in a large decline in yield or even crop failure. Therefore, the current climate variability that occurs in the major winter wheat growing areas needs to be considered in breeding efforts.

Our results also indicate that the modification of crop phenology by cultivar change should be reflected in assessments of potential future crop phenology and crop productivity. Although effects of future breeding strategies on crop phenology are difficult to project, introducing a range of different PVTTs, for example by probabilistic modeling, could be a possible solution.

Further research is required to test the effects of cultivar change on the length of the subsequent reproductive phase of winter wheat. Shortening the vegetative phase may not necessarily translate to a decline in the length of the entire growing season of new winter wheat cultivars. Alternatively, shortening the vegetative period could also be compensated by an increase in the length of the reproductive period. The length of the reproductive phases of new cultivars of winter rapeseed and maize for example, have significantly increased during the past 30 years in Germany^24^ and China^25^, respectively. The trend detected for the day of yellow ripeness (−0.31 days per year) of winter wheat was more negative than the trend detected for the heading day (−0.20 days per year) for Germany and the period from 1951–2004^17^. The length of the vegetative phase of different rice cultivars declined, whereas the reproductive phase was prolonged in the period of 1981–2009 in China^26^. Therefore, it is not recommended to assume that our findings for the vegetative phase can directly be transferred to the generative phase.

Although our results clearly showed that winter wheat cultivars released in the past four decades in Germany were adjusted toward earlier heading and flowering, the underlying physiological relationships that explain these changes and their interactions with the environment remain unknown. In general, the factors considered include vernalization requirements and effectiveness, sensitivity to photoperiod, sensitivity of the development rate to temperature or a combination of these factors. Identifying the reasons for the changes in the phenological characteristics of the cultivars (due to changes in vernalization requirements and sensitivity, photoperiod sensitivity or temperature requirements or combinations of these factors) and the genetic modifications required to implement these changes is not an objective of this study. Identifying this information would require additional data and different methods and could be the objective of subsequent research. It is also not clear whether the detected changes in cultivar properties are an intended or unintended response to the already changing climate or whether the phenological properties of the cultivars have been modified as a result of breeding towards other common targets such as better grain quality or higher resistance against pests and diseases. Finally, a better understanding is required to determine the extent that our results can be generalized for other crops.

## Methods

### Field experiments

In 1904, a static long-term NKPCa fertilization field experiment was established at the Dikopshof near Cologne, Germany (50°48′ 21″ North, longitude: 6°59′ 9″ East, altitude: 62 m), which is characterized by an Atlantic climate with mild winters and summers, a mean annual temperature of 10.5 °C and a mean annual precipitation of 688 mm (1951–2015). The very fertile soil is a humous fine sandy loam formed from loess with a depth of approximately 100 cm. The Haplic (Chromic) Luvisol developed from the loess layer over a sandy-stony, highly permeable Pleistocene middle terrace of the Rhine River^27^. The plant available water capacity and the field capacity (0–100 cm) of the soil are approximately 230 mm and 310 mm, respectively^28^. The winter wheat cultivars received sufficient amounts of crop nutrients from synthetic fertilizer (120 kg N ha^−1^, 31 kg P ha^−1^, 116 kg K ha^−1^). Twelve cultivars were selected based on annual reports of the long-term experiment Dikopshof from 1945 to 2015 (Supplementary Table 1), representing cultivars typical of the region that were grown as part of the long-term experiment. Crop phenology observations were conducted for the period from sowing to the end of flowering with observations taken at two-day intervals for the period around the heading day in the 2015/2016 and 2016/2017 growing seasons. Phenology was recorded according to the BBCH scale^29^. Obvious visual differences in the phenological stages of the old and new cultivars were observed (Supplementary Figure 4). We observed ten plants in the center of each plot that contained 11 rows of plants without replication, and whenever the stamens appeared in more than five plants, the date was recorded as the end of flowering.

### Processing of long-term phenology data

Phenology observations of winter wheat, including the beginning of emergence and beginning of heading, were obtained from the phenological observation network of the German Meteorological Service^30^ for the period from 1951 to 2013. Mean values and standard deviations for the phenological stages were calculated for each of the 86 eco-regions in Germany to filter out potential outliers^31^. We restricted our analysis to 8 federal states in western Germany because the time series of observations was incomplete for the other states. The total number of observations after applying the filtering process was 495,219 from 4824 sites across the study region. We interpolated phenology observations for each year to a 1 km × 1 km grid using a modified inverse distance weighting method and accounted for the spatial variability in temperature and day length between the observed points and the corresponding effect on the spatial pattern of the phenological stages^16^. The spatial interpolation was applied to reduce the effects caused by the heterogeneity in the spatial and temporal distribution of the observations to obtain uniform data coverage across time and space. There were only a few stations that had complete data coverage for all the phenological stages and the whole study period. A cropland mask, which was based on the 2006 CORINE land-cover classification^32^, was then applied to the 1 km × 1 km grids to mask out areas with natural vegetation, forests or grasslands, which are often located in mountainous regions.

### Processing of temperature data

The daily mean temperature for more than 1100 weather stations and interpolated grids of monthly mean temperatures at a 1 km × 1 km resolution for the period from 1951 to 2013 were obtained from the WebWerdis portal of the German Meteorological Service^15^. The daily mean temperature was computed for each 1 km × 1 km grid cell and for each day of the period from 1951 to 2013^15^. Similar to the processing of the phenological data, the cropland mask was applied to the 1 km × 1 km daily temperature grids, and any subsequent analyses were constrained to the grid cells containing cropland.

### Phenology model

We employed a winter wheat phenology model using the modeling platform SIMPLACE^15,33^ to calculate the PVTT required between the observed days of emergence and heading. The crop phenology component of the model (SIMPLACE < LINTUL2>) was used to consider vernalization and photoperiod effects on winter wheat phenology^34^. In the model, the crop development rate in the period between emergence and heading was relative to the daily increment of PVTT (°C day). The PVTT represented a cultivar-specific constant reflecting the temperature sum above a specific base temperature, corrected for the effect of vernalization and photoperiod, required for crop development between specific phenological stages:

1 $$PVTT=\sum _{i=1}^{N}{T}_{ef{f}_{i}}\times {P}_{{f}_{i}}\times {V}_{{f}_{i}}$$

where ${T}_{ef{f}_{i}}$ is the effective temperature on day *i*, ${P}_{{f}_{i}}$ is the photoperiod factor for day *i*, ${V}_{{f}_{i}}$ is the vernalization factor for day *i*, and N is the length of the phenological phase in days. The daily increment in the effective temperature ${T}_{ef{f}_{i}}$ was calculated as the sum of the daily temperatures ${T}_{mea{n}_{i}}$ above a base temperature *T*_*base*_ set to 1 °C and accounted for reduced increments on days with very high mean temperatures^35^:

2 $${T}_{eff}=\{\begin{array}{lcc}{T}_{eff} & if & {T}_{base}\le {T}_{mean}\le {T}_{lim}\,\\ \frac{({T}_{upp}-{T}_{mean})({T}_{lim}-{T}_{base})}{({T}_{upp}-{T}_{lim})} & if & {T}_{lim} < {T}_{mean}\le {T}_{upp}\\ 0 & else & \end{array}$$

with thresholds set to 32 °C for *T*_*lim*_ and 40 °C for *T*_*upp*_. However, daily mean temperatures higher than 32 °C were not observed in western Germany for the period considered in this study^36^. The photoperiod factor ${P}_{{f}_{i}}$ was 0 for the days with a photoperiod less than the base photoperiod set to 7 h d^−1^, 1 for days with a photoperiod longer than the saturated photoperiod set to 17 h d^−1^ and linearly interpolated between 0 and 1 for the days with a photoperiod between 7 and 17 h d^−1^ (Supplementary Figure 5). The vernalization factor ${V}_{{f}_{i}}$ was determined according to the accumulated vernalized days and was linearly interpolated between 0 and 1 for the values of the accumulated vernalized days between 0 and 30 and set to 1 when the threshold of 30 vernalized days was surpassed. The daily increment in accumulated vernalized days was 0 for days with a *T*_*mean*_ less than −4 °C or days with a *T*_*mean*_ larger than 17 °C, 1 for days with a *T*_*mean*_ between 4 °C and 10 °C and linearly interpolated between 0 and 1 for days with temperatures between −4 °C and 4 °C or 10 °C and 17 °C, respectively (Supplementary Figure 5). The model parameters including the base and saturated photoperiods and the parameters for vernalization process (Supplementary Figure 5) were set based on German-wide studies^37,38^. These parameters have been used as standard values for German winter wheat for some decades, and we assumed that these parameters were valid because phenology simulations have been shown to be relatively precise for different locations and years^39^.

To test the accuracy of the model outputs, the normalized root mean squared Error (NRMSE) was calculated between the observed heading day and simulated heading day:

3 $$NRMSE( \% )=\frac{\sqrt{{\sum }_{i=1}^{n}{({S}_{i}-{O}_{i})}^{2}/n}}{\overline{sd}}\times 100$$

where *S*_*i*_ and *O*_*i*_ are the simulated and observed values, respectively, $\overline{sd}$ is the standard deviation of the observations, and *n* is the number of observations. In model runs with the cultivar-specific PVTT, the PVTT in a specific year was derived from the trend line of the piecewise linear regression of the PVTT calculated for the cultivars released in different years and grown in the field experiment (Fig. 2a). In model runs with a fixed PVTT, the PVTT was calculated by averaging the PVTT calculated for the cultivars included in the field experiment. The PVTT from emergence to heading was the only parameter that we adjusted to represent cultivar differences. All other parameters in the phenology model reflecting sensitivity to vernalization and photoperiod were not changed.

### Model application

At the first step of the modeling experiment, we calculated the PVTT_flowering_ and PVTT_heading_ of specific cultivars using the observations from the field experiment (2015/2016 and 2016/2017) and the regional phenology observations (1952–2013), respectively. The trends in both time series were compared to determine whether the cultivars grown in the field experiment were representative of the phenological characteristics of the cultivars used historically in western Germany. Next, we modeled the heading day with different model scenarios to separate the effects of management decisions (changing sowing date and cultivars) on crop phenology from the effects of climate change on phenology. We assumed a difference in the PVTT of 60 °C day as the required temperature sum from heading to flowering to convert between the beginning of flowering simulated with the phenology model and the beginning of heading reported in the variety trials conducted by the Federal Plant Variety Office (Bundessortenamt). To analyze the sole effect of climate on crop phenology, we ran the model with a fixed emergence date and fixed PVTT_flowering_ (average PVTT of all cultivars) obtained from the observations in the field experiment. The combined effects of the climate and sowing date on crop phenology were assessed by executing the model using year- and grid-specific emergence dates and fixed PVTT_flowering_. Finally, the combined effects of climate change, sowing date and cultivars on crop phenology were evaluated using the model with the year- and grid-specific emergence date and cultivar- specific PVTT_flowering_ for each single grid cell (1 km × 1 km). The year- and grid-specific emergence date was prepared based on the observations for each single grid cell and year at a 1 km × 1 km resolution. The cultivar-dependent PVTT_flowering_ for each year was obtained from the trend in the PVTT_flowering_ of the field experiment (Fig. 2a).

### Model validation and sensitivity analysis

The cultivar-specific PVTT_flowering_ was calculated for the first and second year of the field experiment separately by keeping the other parameters of the phenology model constant (Supplementary Figure 5). To test how well this approach can reproduce observed flowering days, a cross-validation model was carried out by simulating the cultivar specific flowering day in the specific growing seasons by using the cultivar-specific PVTT_flowering_ calculated for the other year and comparing the simulated flowering day with the observed flowering day.

A sensitivity analysis was conducted to test how well the differences among the cultivars can be reproduced by adjusting parameters related to either temperature and photoperiod or the vernalization requirements. The required PVTT, base photoperiod and maximum number of vernalization days were selected as target parameters. Consequently, three sets of parameters including fixed photoperiod and vernalization but cultivar-specific thermal requirement (PVTT_T), fixed vernalization and thermal requirement but cultivar-specific base photoperiod (PVTT_P) and fixed photoperiod and thermal requirement but cultivar-specific vernalization days (PVTT_V) were employed for each cultivar to test the sensitivity of the cultivars to temperature, photoperiod and vernalization (Supplementary Table 2).

### Trend analysis

Trends in the timing of the observed and simulated phenological stages, observed PVTT and spring temperature (March-June) were analyzed by a segmented, piecewise linear regression^40^, because previous research identified different rates of change in temperature and crop phenology for the years before the 1970s and the period after the 1970s for the region in this study^3,16,17^. One single break point was identified in the time series by maximizing the combined R^2^ of the two linear regression estimates before and after the breakpoint^41^. The trend in the vegetative period length was analyzed via a simple linear regression due to the high inter-annual variability in the observations. To avoid the strong inter-annual variability in crop phenology resulting in artificial break points close to the beginning or the end of the study period, we only accepted break points in the period from 1961 to 2004^24^. The trend analysis of the mean temperature was restricted to the spring period, because this period is considered the phase when the vernalization requirement of winter wheat is fulfilled, the photoperiod is less limiting, and the crop development rates are therefore particularly sensitive to daily temperature.

## Electronic supplementary material

Supplementary Information
