# Supplementary material for: Climate change effect on wheat phenology depends on cultivar change
Source: Sci Rep. 2018 Mar 20;8:4891. doi: 10.1038/s41598-018-23101-2 (PMC5861073; doi:10.1038/s41598-018-23101-2)
Supplement: Supplementary file 1 — Supplementary Information [file 41598_2018_23101_MOESM1_ESM.pdf]

Supplementary Information

Climate change effect on wheat phenology depends on cultivar change

Ehsan Eyshi Rezaei[1,2,4]*, Stefan Siebert[1,4], Hubert Hüging[1] and Frank Ewert[1,3]

[1] Institute of Crop Science and Resource Conservation, University of Bonn, Katzenburgweg 5, D-53115 Bonn, Germany

[2] Center for Development Research (ZEF), Walter-Flex-Straße 3, 53113 Bonn, Germany

[3] Leibniz Centre for Agricultural Landscape Research, Institute of Landscape Systems Analysis, D-15374 Müncheberg, Germany

[4] Department of Crop Sciences, University of Göttingen, Von-Siebold-Strasse 8, 37075 Göttingen, Germany

*Corresponding author

ehsan.eyshi-rezaei@uni-goettingen.de

Phone: +49(0) 551-39-24676

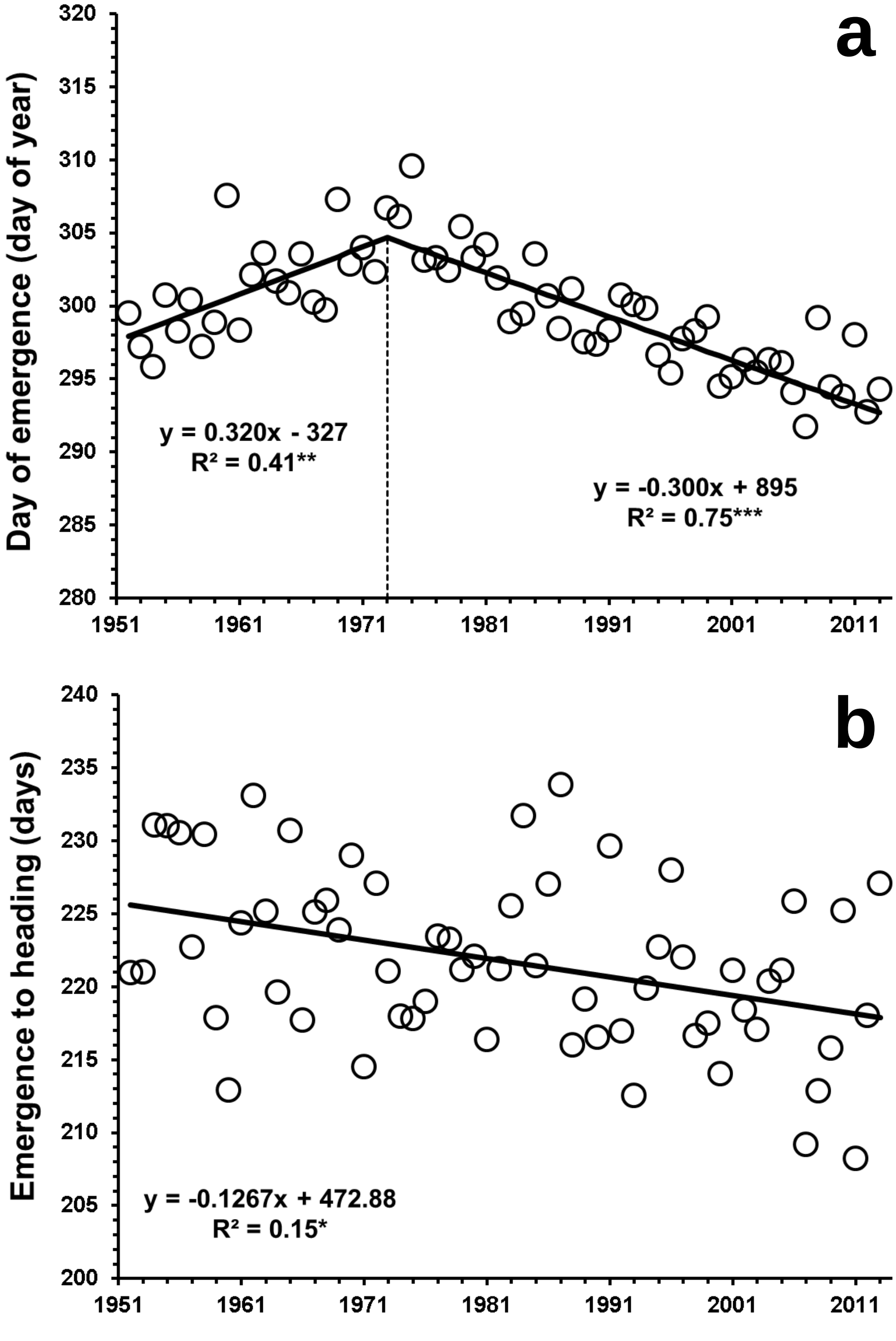

Supplementary Figure 1. Long-term trends in day of emergence (a) and length of vegetative phase (emergence-heading) (b) in the period 1952-2013 at 1 km × 1 km resolution across western Germany.

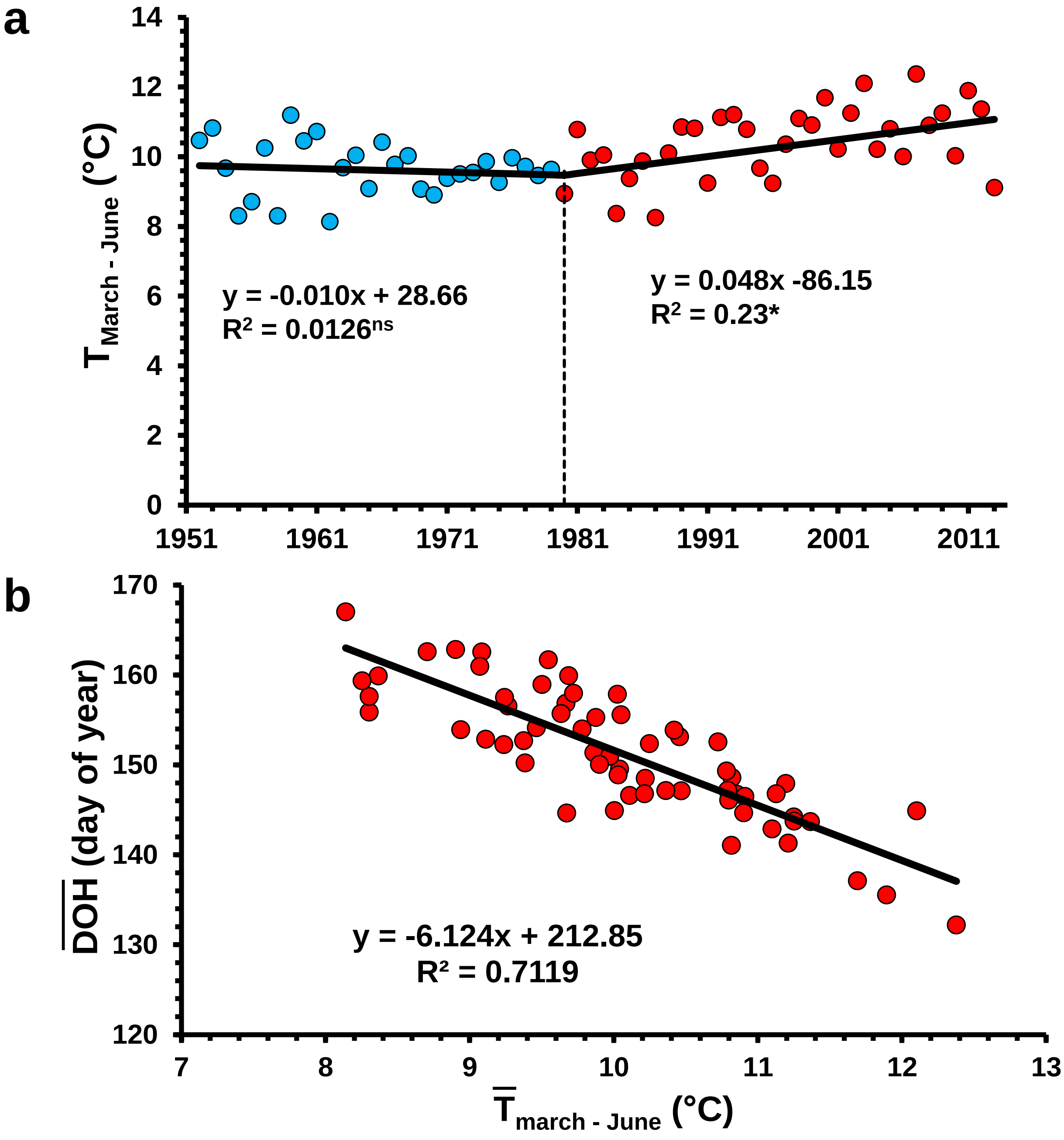

Supplementary Figure 2. The trends in mean temperature (March-June) in the period 1952-2013 across western Germany (a) and the relationship between observed mean heading day and mean temperature (b).

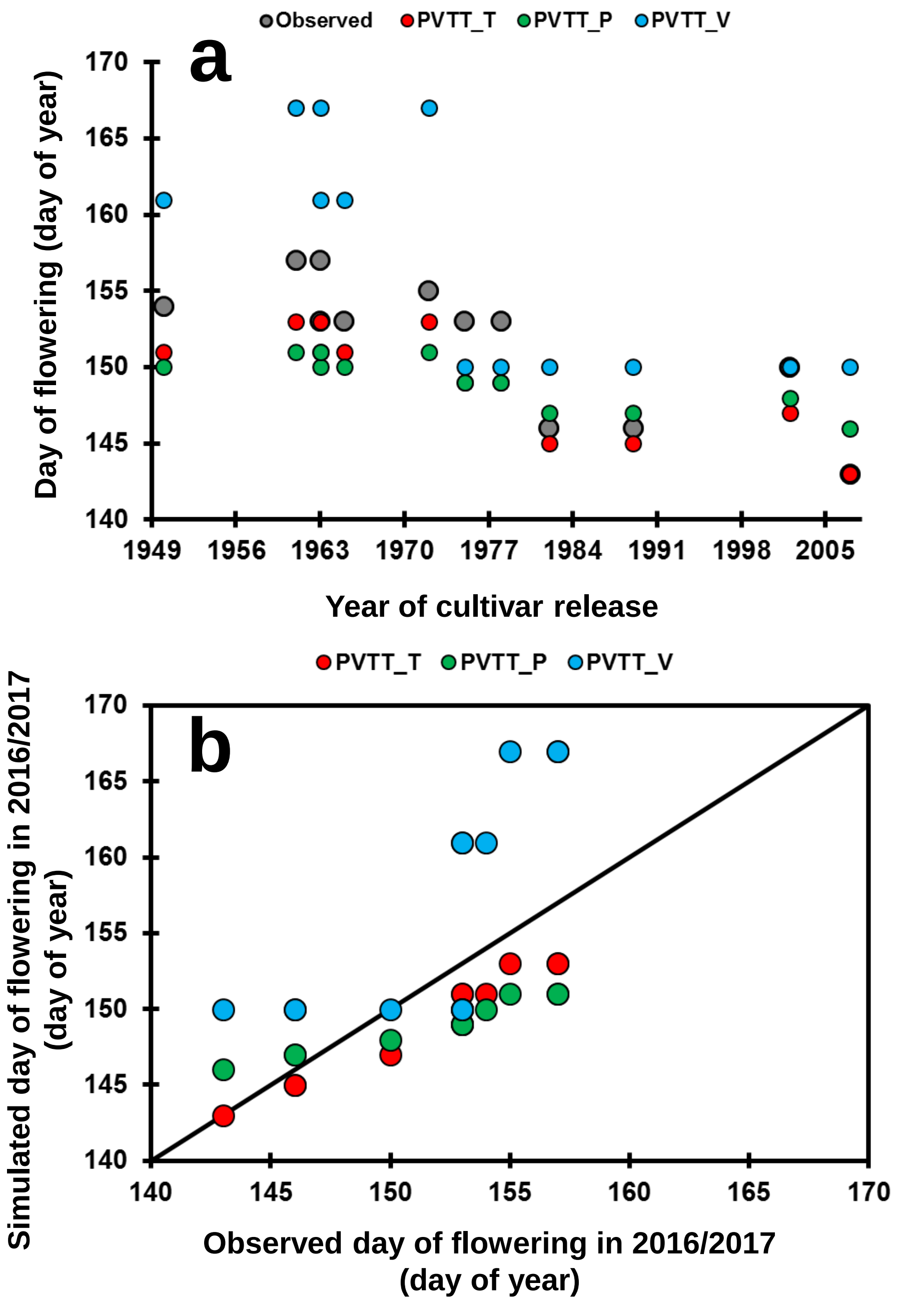

Supplementary Figure 3. Comparison of the observed flowering day in the 2016/2017 growing season to flowering day simulated for the same season with different parameters obtained using the observations for the 2015/2016 growing season to adjust the phenology model parameters. Phenology parameter sets included fix photoperiod and vernalization but cultivar specific thermal requirement (PVTT_T), fix vernalization and thermal requirement but cultivar specific photoperiod (PVTT_P) and fix photoperiod and thermal requirement but cultivar specific

vernalization requirement (PVTT_V) for 12 cultivars representative for distinct historical time periods in the field experiment at Dikopshof, Germany (a). The 1:1 plot of the flowering day simulated for season 2016/2017 with PVTT_T, PVTT_P and PVTT_V parameter sets obtained by using the observations made for season 2015/2016 against flowering day observed for season 2016/2017 (b).

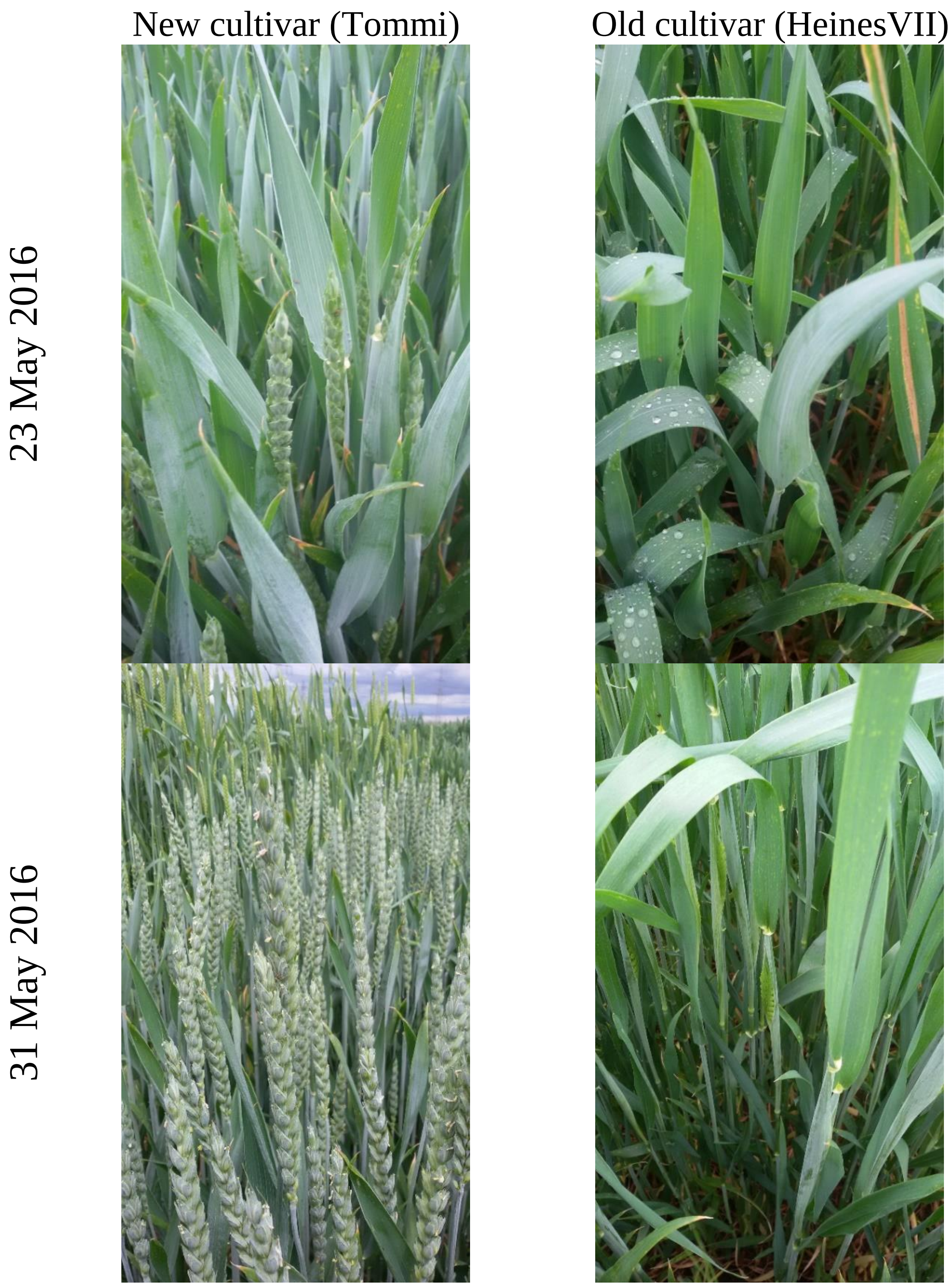

Supplementary Figure 4. The development stages of a new and an old cultivars at two dates in the field experiment.

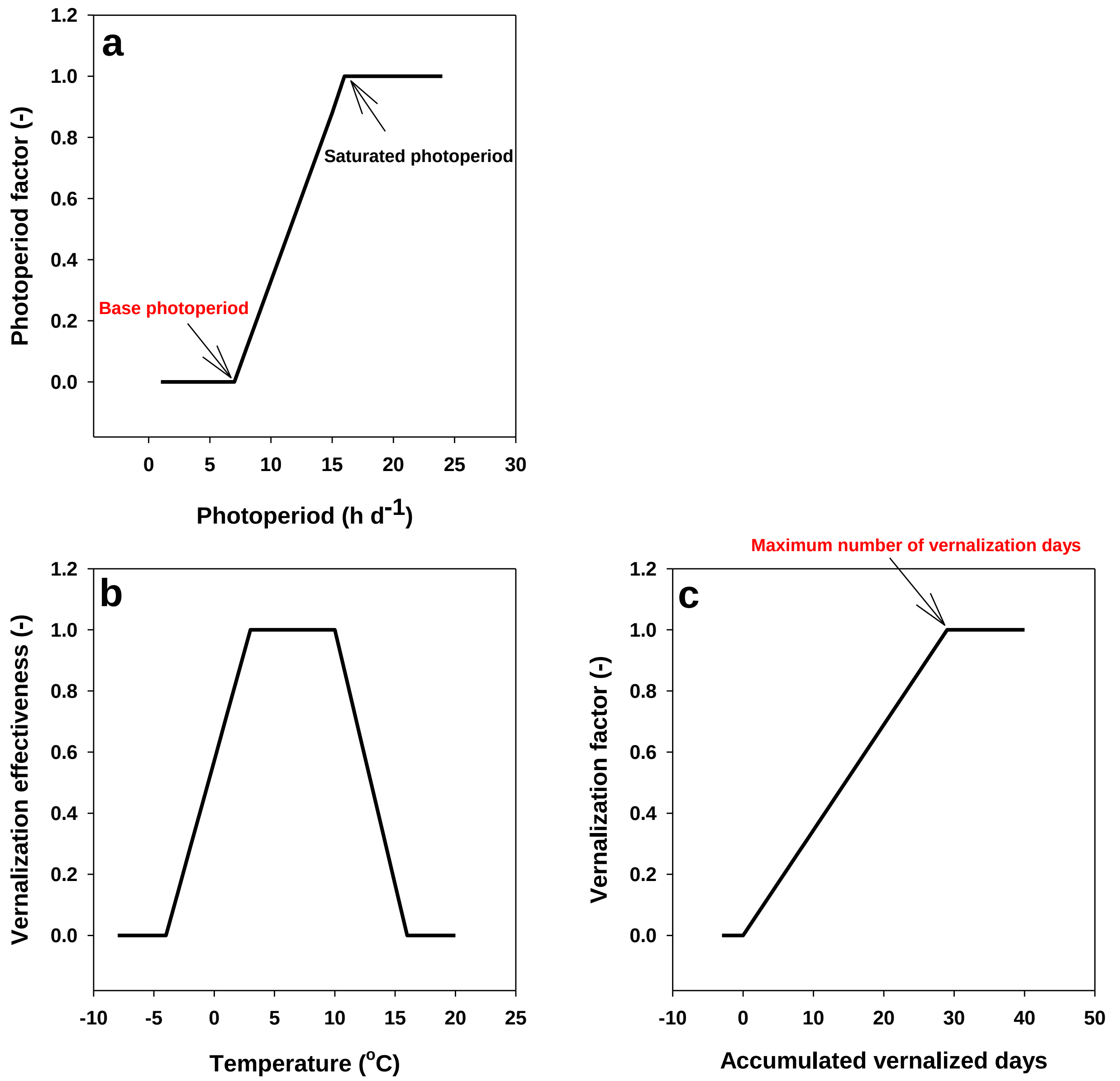

Supplementary Figure 5. Parameter values for effect of photoperiod (a) and vernalization (b and c) on phenological development of winter wheat in the SIMPLACE modeling platform. The parameters highlighted in red are the one that we change at the sensitivity analysis.

Supplementary Table 1. List of representative cultivars cultivated in the field experiment at Dikopshof, Germany, during the 2015/2016 and 2016/2017 growing seasons reporting year of release, observed flowering day and a link to the genetic resources information available in the Genetic Resources Information System for Wheat and Triticale (GRIS).

Cultivar	Year of release*	Flowering in 2016 (day of year)	Flowering in 2017 (day of year)	Web link to GIRS
Heines VII	1950	155	154	https://goo.gl/qBTpcg
Jubilar	1961	157	157	https://goo.gl/6PihcZ
Anda	1963	157	153	https://goo.gl/rFZfjk
Hanno	1963	155	157	https://goo.gl/1M48DA
HS Burgweizen	1965	155	153	https://goo.gl/BBzaFN
Poppelsdorfer Begrannter	1972	157	155	https://goo.gl/7X1hDF
Carimulti	1975	152	153	https://goo.gl/AEBKFY
Okapi	1978	152	153	https://goo.gl/5RLEw2
Sperber	1982	148	146	https://goo.gl/7a8DGb
Greif	1989	148	146	https://goo.gl/2cYAti
Tommi	2002	150	150	https://goo.gl/xgcyzF
Premio	2007	146	143	https://goo.gl/kkmnBK

*Obtained from http://www.wheatpedigree.net/

Supplementary Table 2. List of the parameters for photo vernal thermal time (PVTT), base photoperiod (Pb) and vernalization days (Vdays) used in the sensitivity analysis for the parameter sets with fix photoperiod and vernalization but cultivar specific thermal requirement (PVTT_T), fix vernalization and thermal requirement but cultivar specific photoperiod (PVTT_P) and fix photoperiod and thermal requirement but cultivar specific vernalization requirement (PVTT_V) for 12 cultivars representative for distinct historical time periods used in the field experiment at Dikopshof, Germany.

		Parametrization procedure		
Cultivar/year of release	Parameter	PVTT_T	PVTT_P	PVTT_V
	PVTT	681	660	660
Heines VII/1950	P_b	7	7.2	7
	V_{days}	30	30	190
	PVTT	711	660	660
Jubilar/1961	P_b	7	7.6	7
	V_{days}	30	30	210
	PVTT	681	660	660
Anda/1963	P_b	7	7.2	7
	V_{days}	30	30	190
	PVTT	711	660	660
Hanno/1963	P_b	7	7.6	7
	V_{days}	30	30	210
	PVTT	681	660	660
HS Burgweizen/1965	P_b	7	7.2	7
	V_{days}	30	30	190
	PVTT	711	660	660
Poppelsdorfer Begrannter/1972	P_b	7	7.6	7
	V_{days}	30	30	210
	PVTT	638	660	660
Carimulti/1975	P_b	7	6.6	7
	V_{days}	30	30	1
	PVTT	638	660	660
Okapi/1978	P_b	7	6.6	7
	V_{days}	30	30	1
	PVTT	577	660	660
Sperber/1982	P_b	7	5.5	7
	V_{days}	30	30	1
	PVTT	577	660	660
Greif/1989	P_b	7	5.5	7
	V_{days}	30	30	1
	PVTT	607	660	660
Tommi/2002	P_b	7	6.0	7
	V_{days}	30	30	1

	PVTT	554	660	660
Premio/2007	P_b	7	4.9	7
	V_{days}	30	30	1

PVTT: photo vernal thermal time (°C day), Pb: base photoperiod (h), Vdays: maximum number of vernalization days (d)
